# Drain versus no drain in elective open incisional hernia operations: a registry-based analysis with 39,523 patients

**DOI:** 10.1007/s10029-023-02862-4

**Published:** 2023-08-18

**Authors:** M. Sahm, M. Pross, M. Hukauf, D. Adolf, F. Köckerling, R. Mantke

**Affiliations:** 1 Clinic for General and Visceral Surgery, Brandenburg Medical School, Hochstraße 29, 14770 Brandenburg, Germany; 2 Faculty of Health Sciences Brandenburg, Brandenburg Medical School, Nicolaiplatz 19, 14770 Brandenburg, Germany; 3 Department of Surgery, DRK Kliniken Berlin Köpenick, Salvador Allende Str. 2-8, 12557 Berlin, Germany; 4 StatConsult GmbH, Am Fuchsberg 11, 39112 Magdeburg, Germany; 5 Hernia Center, Vivantes Humboldt Hospital, Academic Teaching Hospital of Charité, University Medicine, Am Nordgraben 2, 13509 Berlin, Germany

**Keywords:** Drain, Open incisional hernia repair, Outcome, Elective hernia operations

## Abstract

**Purpose:**

Elective open incisional hernia operations are a frequently performed and complex procedure. Prophylactic drainage is widely practised to prevent local complications, but nevertheless the benefit of surgical drain placement remains a controversially discussed subject. Objective of this analysis was to evaluate the current status of patient care in clinical routine and outcome in this regard.

**Methods:**

The study based on prospectively collected data of the Herniamed Register. Included were all patients with elective open incisional hernia between 1/2005 and 12/2020 and completed 1-year follow-up. Multiple linear and logistic regression analysis was performed to assess the relation of individual factors to the outcome variables.

**Results:**

Analysed were data from 39,523 patients (28,182 with drain, 11,341 without). Patients with drain placement were significantly older, had a higher BMI, more preoperative risk factors, and a larger defect size. Drained patients furthermore showed a significant disadvantage in the outcome parameters intraoperative complications, general complications, postoperative complications, complication-related reoperations, and pain at the 1-year follow-up. No significant difference was observed with respect to the recurrent rate.

**Conclusion:**

With 71.3%, the use of surgical drainages has a high level of acceptance in elective open incisional hernia operations. The worse outcome of patients is associated with the use of drains, independent of other influencing factors in the model such as patient or surgical characteristics. The use of drains may be a surrogate parameter for other unobserved confounders.

## Introduction

Surgical drains have a long history in medicine as an integral part of the therapeutic concept [1]. Already since the mid-1800s, the use of drains in gastrointestinal surgery has widely been practised. Lawson Tait, a nineteenth century surgeon, even coined the dictum “When in doubt, drain” [2], but in practice the situation turns out to be much more complex and leaves the decision of drain usage to the surgeon's perception of the overall situation. In open ventral hernia repair, drains are traditionally placed to avoid seroma and hematoma formation by facilitating fluid drainage [3]. The prophylactic placement of drains has, however, aroused much controversy as studies have been published indicating that drains often fail to protect against seromas and may even contribute to infectious complications [4]. Traditional intra-abdominal and subcutaneous drains were also assessed within the context of optimizing perioperative management which began with the fast-track concept of Kehlet in 1995 for colon surgery and the ERAS (enhanced recovery after surgery) management in 2005, and their avoidance was recommended in the case of questionable protective effects [5–7]. But what are the consequences for clinical practice? Already in the past, differences between the current status of research and patient care in clinical routine [8] have been observed, leading to a more differentiated view concerning the interpretation of the respective results. Accordingly, a thorough assessment of the quality of care in hernia surgery within the framework of clinical health services research is a prerequisite that contributes an essential element to the further development of optimized therapies in everyday clinical practice. Based on data from the Herniamed Hernia registry, we evaluated the reality of care in elective open incisional hernia operations, with a particular focus on the utilization of drains in this study.

## Material and methods

We evaluated prospectively collected data from 836 centres in Germany, Austria and Switzerland from the internet-based Herniamed Hernia registry and included operated patients from January 5, 2009 to December 31, 2020 with completed 1-year follow-up visit in this evaluation. The inclusion criteria were elective incisional hernia operations with open procedures (open direct suture, open onlay, open sublay, open intraperitoneal onlay mesh (IPOM), component separation). Exclusion criteria were incompletely documented cases, invalid age information, patients under the age of 16, and the use of non-approved meshes. Senior or high-risk patients were not excluded. All patients signed a consent form agreeing to the processing of their data [9]. Baseline demographic data included age, gender, BMI (body mass index), and ASA (American Society of Anesthesiologists) score. In addition to the surgical methods mentioned above, the use of drains, EHS (European Hernia Society) classification, mesh implantation, pre- and postoperative pain, and recurrences were recorded. Single outcome and influencing variables (risk factors, complications) were summarized as global variables. A general, intra- or postoperative complication or risk factor was considered present if at least one single item applied.

### Plausibility assessment

A plausibility check was performed to confirm the presence of a correct data set with patient master and operation data. Furthermore, the plausibility of length-of-stay data, information on surgery time and mesh size, age, weight, height, BMI, and follow-up data was verified.

### Statistical analysis

All analyses were performed using SAS 9.4 software (SAS Institute Inc., Cary, NC, USA). A p-value of ≤ 0.05 was considered statistically significant. Univariate descriptive statistics were performed for the comparison of drain use (yes vs. no). All categorical patient data are presented in absolute and relative counts, while mean and standard deviation (SD) are shown for continuous data. Unadjusted analyses were carried out to assess the effect of individual influencing variables on an outcome parameter. The Chi-square test was used for categorical target variables, and the robust t-test (Satterthwaite) was used for continuous variables. Multivariable analyses were performed using the binary logistic regression model. All pair-wise odds ratios are given with the corresponding 95% confidence intervals. To rule out a potential bias in the selection of the analysis population (patients with 1-year follow-up) compared to patients without follow-up, standardized differences were estimated for the two populations.

## Results

### Patient and operation characteristics

**Fig. 1:**
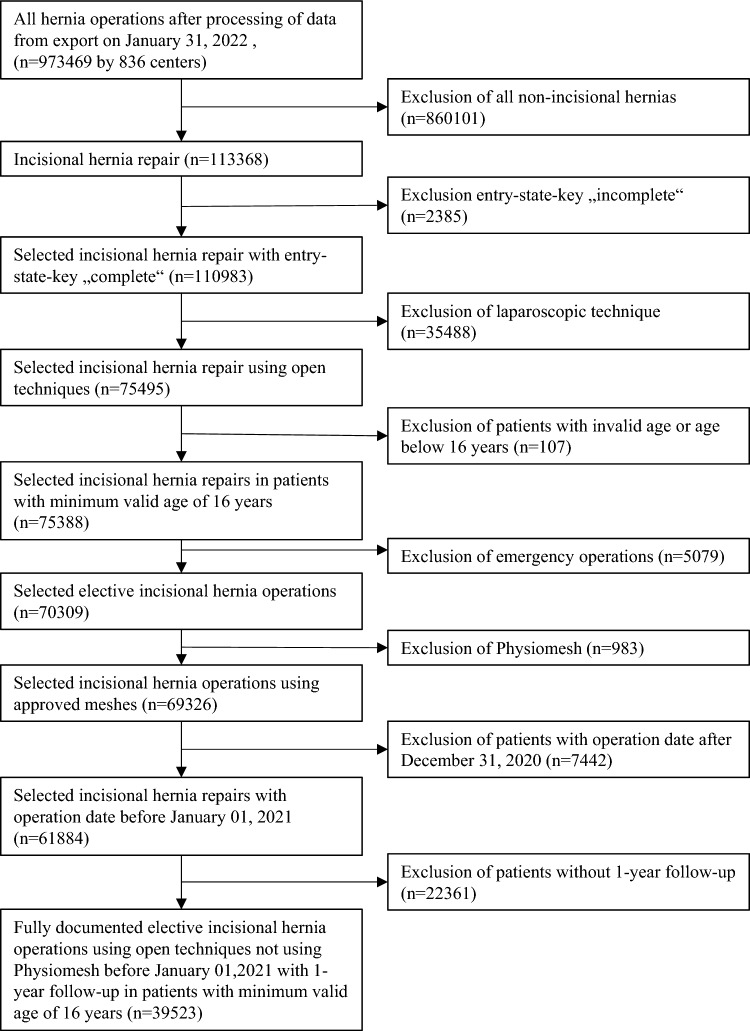
Flowchart of patient inclusion

Between January 5, 2009 and December 31, 2020, data from 39,523 patients who underwent elective open incisional hernia surgery with completed 1-year follow-up were entered into the Herniamed Registry (Fig. 1). Drains were used in 28,182 patients (71.31%) undergoing elective surgery, while 11,341 patients (28.69%) did not receive a drain. Drained patients had an average age of 63.6 ± 12.8 years (mean ± SD) and were thus significantly older than patients without drain use who had an average age of 59.8 ± 15.1 years (*p* < 0.001). Additionally, the BMI was significantly higher in patients with compared to patients without drains (29.8 ± 5.9 vs. 27.9 ± 5.4, *p* < 0.001) (Table 1).

**Table 1:** Unadjusted analysis results for homogeneity between drain use (yes vs. no) and age and BMI, respectively; descriptive statistics and results of unadjusted analysis for homogeneity between the comparison groups (drainage yes vs. no) and categorical influencing variables

		Drainage				
		Yes		No		*p*
		*n*	Mean ± SD	*n*	Mean ± SD	
Age (years)		28,182	63.6 ± 12.8	11,341	59.8 ± 15.1	< 0.001
BMI (kg/m^2)^		28,080	29.8 ± 5.9	11,305	27.9 ± 5.4	< 0.001
		*n*	%	*n*	%	
Gender	Male	14,377	51.0	5737	50.6	0.441
	Female	13,805	49.0	5604	49.4	
ASA	I	2261	8.0	2102	18.5	< 0.001
	II	15,539	55.1	6434	56.7	
	III/IV	10,382	36.8	2805	24.7	
Operation technique	Open—onlay	2324	8.2	518	4.6	< 0.001
	Open—sublay	18,748	66.5	3693	32.6	
	Open—IPOM	4004	14.2	3122	27.5	
	Component separation	1441	5.1	163	1.4	
	Open—direct suture	1665	5.9	3845	33.9	
Defect size	I (< 4 cm)	6365	22.6	7696	67.9	< 0.001
	II (4–10 cm)	14,991	53.2	2904	25.6	
	III (> 10 cm)	6826	24.2	741	6.5	
EHS classification	Medial	21,913	77.8	8672	76.5	< 0.001
	Lateral	4039	14.3	1921	16.9	
	Combined	2230	7.9	748	6.6	
Preoperative pain	No	9415	33.4	3599	31.7	0.006
	Yes	16,433	58.3	6777	59.8	
	Unknown	2334	8.3	965	8.5	
Mesh	Yes	26,191	92.9	7324	64.6	< 0.001
	No	1991	7.1	4017	35.4	
Recurrent operation	Yes	5873	20.8	1904	16.8	< 0.001
	No	22,309	79.2	9437	83.2	
Chronic obstructive pulmonary disease (COPD)	Yes	3227	11.5	977	8.6	< 0.001
	No	24,955	885	10,364	91.4	
Diabetes	Yes	4033	14.3	1025	9.0	< 0.001
	No	24,149	85.7	10,316	91.0	
Aortic aneurysm	Yes	540	1.9	117	1.0	< 0.001
	No	27,642	98.1	11,224	99.0	
Immunosuppression	Yes	599	2.1	217	1.9	0.180
	No	27,583	97.9	11,124	98.1	
Corticoids	Yes	500	1.8	178	1.6	0.156
	No	27,682	98.2	11,163	98.4	
Smoking	Yes	3605	12.8	1372	12.1	0.060
	No	24,577	87.2	9969	87.9	
Coagulopathy	Yes	747	2.7	193	1.7	< 0.001
	No	27,435	97.3	11,148	98.3	
Antithrombotic medication	Yes	3665	13.0	1141	10.1	< 0.001
	No	24,517	87.0	10,200	89.9	
Anticoagulant medication	Yes	969	3.4	286	2.5	< 0.001
	No	27,213	96.6	11,055	97.5	

In the unadjusted analysis of the relationship between the two patient groups (drain vs. no drain) with respect to patient and operation characteristics, the expression of almost all variables differed significantly. Only with respect to gender, no statistically significant difference could be observed (*p* = 0.441) (Table 1). In the detailed evaluation of the unadjusted analyses concerning items relevant for general complications, significant differences between the two patient groups for fever (*p* < 0.001) and pulmonary embolism (p 0.007) were detected. For thrombosis, *p* = 0.059. The unadjusted analysis results of the relationship between postoperative complications and drain use are presented in Table 2. No significant differences in the topic-specific items seroma, wound healing disorder, and infection were observed (*p* < 0.001 each).

**Table 2:** Descriptive statistics and results of unadjusted analysis for homogeneity between comparison groups (drainage yes vs. no) and outcome variables as well as individual items of postoperative complications

		Drainage				
		Yes		No		*p*
		*n*	%	*n*	%	
Intraoperative complications—total	Yes	551	2.0	94	0.8	< 0.001
	No	27,631	98.0	11,247	99.2	
General complications—total	Yes	1269	4.5	220	1.9	< 0.001
	No	26,913	95.5	11,121	98.1	
Fever	Yes	139	0.5	21	0.2	< 0.001
	No	28,043	99.5	11,320	99.8	
Pulmonary embolism	Yes	53	0.2	8	< 0.1	0.007
	No	28,129	99.8	11,333	> 99.1	
Thrombosis	Yes	30	0.1	5	< 0.1	0.059
	No	28,152	99.1	11,336	> 99.9	
Postoperative complications—total	Yes	3041	10.8	562	5.0	< 0.001
	No	25,141	89.2	10,779	95.0	
Complication-related reoperations	Yes	1387	4.9	204	1.8	< 0.001
	No	26,795	95.1	11,137	98.2	
Recurrence at 1-year follow-up	Yes	1422	5.0	607	5.4	0.212
	No	26,760	95.0	10,734	94.6	
Pain on exertion at 1-year follow-up	Yes	5494	19.5	1824	16.1	< 0.001
	No	22,688	80.5	9517	83.9	
Pain at rest at 1-year follow-up	Yes	3148	11.2	1008	8.9	< 0.001
	No	25,034	88.8	10,333	91.1	
Pain requiring treatment at 1-year follow-up	Yes	2472	8.8	766	6.8	< 0.001
	No	25,710	91.2	10,575	93.2	
Bleeding	Yes	748	2.7	138	1.2	< 0.001
	No	27,434	97.3	11,203	98.8	
Seroma	Yes	1353	4.8	255	2.2	< 0.001
	No	26,829	95.2	11,086	97.8	
Prolonged ileus or obstruction	Yes	188	0.7	30	0.3	< 0.001
	No	27,994	99.3	11,311	99.7	
Bowel injury/anastomotic insufficiency	Yes	77	0.3	25	0.2	0.349
	No	28,105	99.7	11,316	99.8	
Wound healing disorder	Yes	889	3.2	142	1.3	< 0.001
	No	27,293	96.8	11,199	98.7	
Infection	Yes	478	1.7	75	0.7	< 0.001
	No	27,704	98.3	11,266	99.3	

### Intraoperative complications in logistic regression analyses

The risk of intraoperative complications was significantly associated with defect size, surgical procedure, drain use, age (*p* < 0.001 each), and recurrence (*p* = 0.006). Specifically, intraoperative complications occurred more frequently in larger defects, the surgical procedures open-direct suture and open-IPOM, drained patients (OR odds ratio = 1902 [1483; 2438]), elderly patients, and patients with recurrences (Table 3).

**Table 3:** Results of the multivariable analysis for intraoperative complications including odds ratios with corresponding 95% confidence interval

Variable	*p*-value	Categories	Odds ratio	LCL	UCL	*p*-value (pair-wise)
Defect size	< 0.001	III (> 10 cm) vs. I (< 4 cm)	4.905	3.669	6.556	< 0.001
		II (4–10 cm) vs. I (< 4 cm)	3.261	2.501	4.253	< 0.001
		III (> 10 cm) vs. II (4–10 cm)	1.504	1.258	1.798	< 0.001
Operation technique	< 0.001	Open—direct suture vs. Open—sublay	4.097	2.414	6.954	< 0.001
		Open—IPOM vs. open—sublay	1.570	1.272	1.936	< 0.001
		Open—direct suture vs. open—onlay	3.344	1.854	6.031	< 0.001
		Open—direct suture vs. component separation	3.054	1.667	5.593	< 0.001
		Open—direct suture vs. open—IPOM	2.610	1.519	4.487	< 0.001
		Component separation vs. open—sublay	1.342	0.962	1.871	0.083
		Open—IPOM vs. open—onlay	1.281	0.907	1.809	0.160
		Open—onlay vs. open—sublay	1.225	0.892	1.682	0.209
		Component separation vs. open—IPOM	0.855	0.598	1.222	0.389
		Component separation vs. open—onlay	1.095	0.710	1.688	0.681
Drainage	< 0.001	Yes vs. no	1.902	1.483	2.438	
Age [10-years-OR]	< 0.001		1.166	1.088	1.250	
Recurrent operation	0.006	Yes vs. no	1.293	1.075	1.554	
BMI [5-points-OR]	0.120		1.056	0.986	1.130	
Risk factors	0.296	Yes vs. no	1.092	0.926	1.287	
EHS classification	0.473	Medial vs. lateral	1.158	0.912	1.471	0.227
		Lateral vs. combined	0.857	0.609	1.205	0.374
		Medial vs. combined	0.992	0.755	1.305	0.956
Gender	0.486	Female vs. male	1.059	0.902	1.242	
ASA	0.851	II vs. I	1.092	0.781	1.527	0.606
		III/IV vs. I	1.064	0.742	1.525	0.736
		III/IV vs. II	0.974	0.818	1.161	0.770
Preoperative pain	0.900	Yes vs. no	0.985	0.830	1.169	0.860
		Unknown vs. no	0.930	0.682	1.268	0.646
		Yes vs. unknown	1.059	0.787	1.425	0.707
Mesh	0.998	Yes vs. no	1.001	0.600	1.671	

### General complications in logistic regression analyses

The general complications were significantly related to defect size, EHS classification (lateral), the need for drains (*p* < 0.001 each), and tendentially also BMI (*p* = 0.077). The risk of general complications was increased by larger defects, higher ASA score, older age, the presence of risk factors, component separation, and drain use (*OR* = 1421 [1209; 1670]) (Table 4).

**Table 4:** Results of the multivariable analysis for general complications including odds ratios with corresponding 95% confidence interval

Variable	*p*-value	Categories	Odds ratio	LCL	UCL	*p*-value (pair-wise)
Defect size	< 0.001	III (> 10 cm) vs. I (< 4 cm)	2.862	2.397	3.418	< 0.001
		III (> 10 cm) vs. II (4–10 cm)	1.633	1.449	1.841	< 0.001
		II (4–10 cm) vs. I (< 4 cm)	1.752	1.486	2.067	< 0.001
ASA	< 0.001	III/IV vs. II	1.582	1.409	1.776	< 0.001
		III/IV vs. I	1.952	1.478	2.578	< 0.001
		II vs. I	1.234	0.943	1.614	0.125
Age [10-years-OR]	< 0.001		1.197	1.141	1.256	
Risk factors	< 0.001	Yes vs. no	1.429	1.279	1.596	
Operation technique	< 0.001	Component separation vs. open—sublay	1.684	1.389	2.041	< 0.001
		Component separation vs. open—onlay	2.177	1.632	2.903	< 0.001
		Component separation vs. open—IPOM	1.581	1.271	1.968	< 0.001
		Open—direct suture vs. component separation	0.466	0.300	0.724	< 0.001
		Open—IPOM vs. open—onlay	1.376	1.065	1.778	0.015
		Open—onlay vs. open—sublay	0.774	0.612	0.977	0.031
		Open—direct suture vs. open—IPOM	0.737	0.486	1.117	0.150
		Open—direct suture vs. open—sublay	0.785	0.522	1.179	0.243
		Open—IPOM vs. open—sublay	1.065	0.922	1.230	0.394
		Open—direct suture vs. open—onlay	1.014	0.644	1.598	0.951
EHS classification	< 0.001	Medial vs. lateral	1.565	1.307	1.875	< 0.001
		Lateral vs. combined	0.619	0.486	0.789	< 0.001
		Medial vs. combined	0.969	0.809	1.161	0.736
Drainage	< 0.001	Yes vs. no	1.421	1.209	1.670	
BMI [5-points-OR]	0.077		1.042	0.996	1.091	
Gender	0.248	Female vs. male	1.065	0.957	1.186	
Mesh	0.272	Yes vs. no	0.817	0.570	1.172	
Preoperative pain	0.455	Yes vs. no	1.077	0.959	1.209	0.210
		Unknown vs. no	1.047	0.852	1.287	0.664
		Yes vs. unknown	1.029	0.845	1.253	0.778
Recurrent operation	0.704	Yes vs. no	1.026	0.900	1.169	

### Postoperative complications in logistic regression analyses

The occurrence of postoperative complications was significantly associated with the defect size, BMI, the presence of risk factors, surgical method, EHS classification, the use of drains, ASA classification, and age (*p* < 0.001 each). A larger defect, a higher BMI, the presence of at least one risk factor, component separation and open-IPOM, the use of drains (*OR* = 1366 [1230; 1517]), a higher ASA score, and older age increased the risk for postoperative complications (Table 5).

**Table 5:** Results of the multivariable analysis for postoperative complications including odds ratios with corresponding 95% confidence interval

Variable	*p*-value	Categories	Odds ratio	LCL	UCL	*p*-value (pair-wise)
Defect size	< 0.001	III (> 10 cm) vs. I (< 4 cm)	2.592	2.314	2.903	< 0.001
		III (> 10 cm) vs. II (4–10 cm)	1.616	1.489	1.753	< 0.001
		II (4–10 cm) vs. I (< 4 cm)	1.604	1.447	1.779	< 0.001
BMI [5-points-OR]	< 0.001		1.145	1.112	1.180	
Risk factors	< 0.001	Yes vs. no	1.364	1.268	1.468	
Operation technique	< 0.001	Component separation vs. open—IPOM	1.788	1.526	2.094	< 0.001
		Component separation vs. open—sublay	1.496	1.302	1.718	< 0.001
		Component separation vs. open—onlay	1.559	1.294	1.879	< 0.001
		Open—direct suture vs. component separation	0.571	0.416	0.783	< 0.001
		Open—IPOM vs. open—sublay	0.837	0.757	0.925	< 0.001
		Open—IPOM vs. open—onlay	0.872	0.744	1.023	0.093
		Open—direct suture vs. open—sublay	0.853	0.638	1.141	0.285
		Open—direct suture vs. open—onlay	0.890	0.651	1.216	0.463
		Open—onlay vs. open—sublay	0.959	0.835	1.102	0.558
		Open—direct suture vs. open—IPOM	1.020	0.757	1.373	0.897
EHS classification	< 0.001	Medial vs. lateral	1.483	1.321	1.666	< 0.001
		Lateral vs. combined	0.712	0.605	0.838	< 0.001
		Medial vs. combined	1.057	0.931	1.200	0.392
Drainage	< 0.001	Yes vs. no	1.366	1.230	1.517	
ASA	< 0.001	III/IV vs. II	1.212	1.121	1.310	< 0.001
		III/IV vs. I	1.264	1.080	1.480	0.004
		II vs. I	1.043	0.901	1.208	0.571
Age [10-years-OR]	< 0.001		1.059	1.028	1.092	
Recurrent operation	0.164	Yes vs. no	1.063	0.975	1.158	
Mesh	0.265	Yes vs. no	1.163	0.892	1.516	
Preoperative pain	0.465	Yes vs. no	1.048	0.970	1.132	0.235
		Yes vs. unknown	1.040	0.912	1.187	0.557
		Unknown vs. no	1.007	0.878	1.156	0.919
Gender	0.466	Female vs. male	0.974	0.907	1.046	

### Complication-related reoperations in logistic regression analyses

The risk of reoperation was significantly associated with defect size, the presence of risk factors, the use of drains, EHS classification, BMI, surgical method and ASA classification (*p* < 0.001 each). The complication-related reoperation rate was significantly higher when drains were used (*OR* = 1632 [1385, 1924]). In addition, a larger defect, the presence of a risk factor, a higher BMI, component separation, and a higher ASA score also increased the risk of reoperation (Table 6).

**Table 6:** Results of the multivariable analysis for complication-related reoperations including odds ratios with corresponding 95% confidence interval

Variable	*p*-value	Categories	Odds ratio	LCL	UCL	*p*-value (pair-wise)
Defect size	< 0.001	III (> 10 cm) vs. I (< 4 cm)	2.609	2.201	3.093	< 0.001
		III (> 10 cm) vs. II (4–10 cm)	1.553	1.382	1.744	< 0.001
		II (4–10 cm) vs. I (< 4 cm)	1.681	1.436	1.967	< 0.001
Risk factors	< 0.001	Yes vs. no	1.394	1.253	1.551	
Drainage	< 0.001	Yes vs. no	1.632	1.385	1.924	
EHS classification	< 0.001	Medial vs. lateral	1.780	1.479	2.142	< 0.001
		Lateral vs. combined	0.563	0.441	0.719	< 0.001
		Medial vs. combined	1.003	0.840	1.197	0.975
BMI [5-points-OR]	< 0.001		1.115	1.068	1.164	
Operation technique	< 0.001	Component separation vs. open—IPOM	1.774	1.427	2.205	< 0.001
		Component separation vs. open—sublay	1.587	1.316	1.914	< 0.001
		Component separation vs. open—onlay	1.593	1.228	2.067	< 0.001
		Open—direct suture vs. component separation	0.440	0.277	0.697	< 0.001
		Open—direct suture vs. open—sublay	0.698	0.454	1.073	0.101
		Open—direct suture vs. open—onlay	0.700	0.441	1.111	0.131
		Open—IPOM vs. open—sublay	0.895	0.773	1.035	0.135
		Open—direct suture vs. open—IPOM	0.780	0.502	1.211	0.268
		Open—IPOM vs. open—onlay	0.898	0.713	1.131	0.361
		Open—onlay vs. open—sublay	0.996	0.815	1.218	0.970
ASA	< 0.001	III/IV vs. II	1.336	1.193	1.495	< 0.001
		III/IV vs. I	1.347	1.066	1.702	0.013
		II vs. I	1.009	0.809	1.258	0.940
Preoperative pain	0.105	Yes vs. no	1.106	0.987	1.238	0.082
		Unknown vs. no	1.193	0.983	1.447	0.074
		Yes vs. unknown	0.927	0.772	1.113	0.417
Age [10-years-OR]	0.107		1.037	0.992	1.084	
Gender	0.139	Female vs. male	0.924	0.833	1.026	
Recurrent operation	0.194	Yes vs. no	1.085	0.959	1.227	
Mesh	0.812	Yes vs. no	1.047	0.717	1.530	

### Results of the 1-year follow-up in logistic regression analyses

The risk of recurrences at the 1-year follow-up was strongly related to previous recurrences, the surgical method (e.g. open-onlay), EHS classification, higher BMI, larger defect size (*p* < 0.001 each), the use of meshes (*p* = 0.001), the ASA score (*p* = 0.002), female gender (*p* = 0.004), higher age (*p* = 0.031), and preoperative pain (*p* = 0.050). No significant relation could be shown for the use of drains (*p* = 0.650) (Table 7). Pain at rest at the 1-year follow-up was significantly associated with higher age, preoperative pain, female gender, postoperative complications, EHS classification, higher BMI, prior surgeries, drain use, larger defect size, and ASA score (*p* = 0.001 each). The risk of pain at rest increased with drain use (*OR* = 1174 [1075; 1282]) (Table 8). The pain on exertion at the 1-year follow-up was significantly dependent on age, gender, preoperative pain, postoperative complications, EHS classification, defect size, BMI, use of drains, presence of recurrences (*p* = 0.001 each), surgical method (*p* = 0.001), presence of risk factors (*p* = 0.008) and ASA score (*p* = 0.020). Drain use increased the risk of pain on exertion (*OR* = 1.173 [1.094; 1.258]) (Table 9). Pain requiring treatment at the 1-year follow-up was significantly related to age, preoperative pain, gender, postoperative complications, EHS classification, recurrent interventions, ASA score, defect size, use of drains, presence of risk factors (*p* < 0.001 each), BMI (*p* = 0.011), and surgical method (*p* = 0.023). The use of drains was furthermore associated with a higher risk of pain requiring treatment (*OR* = 1211 [1097; 1338]) (Table 10).

**Table 7:** Results of the multivariable analysis for recurrence in the follow-up including odds ratios with corresponding 95% confidence interval

Variable	*p*-value	Categories	Odds ratio	LCL	UCL	*p*-value (pair-wise)
Recurrent operation	< 0.001	Yes vs. no	1.489	1.342	1.652	
Operation technique	< 0.001	Open—onlay vs. open—sublay	1.609	1.369	1.891	< 0.001
		Open—IPOM vs. open—sublay	1.379	1.218	1.561	< 0.001
		Component separation vs. open—onlay	0.608	0.458	0.808	< 0.001
		Open—direct suture vs. open—sublay	1.505	1.120	2.024	0.007
		Component separation vs. open—IPOM	0.710	0.546	0.922	0.010
		Open—direct suture vs. component separation	1.538	1.055	2.243	0.025
		Open—IPOM vs. open—onlay	0.857	0.715	1.026	0.094
		Open—direct suture vs. open—IPOM	1.092	0.808	1.475	0.568
		Open—direct suture vs. open—onlay	0.935	0.680	1.288	0.682
		Component separation vs. open—sublay	0.979	0.762	1.256	0.865
EHS classification	< 0.001	Medial vs. lateral	0.685	0.610	0.769	< 0.001
		Lateral vs. combined	1.305	1.079	1.579	0.006
		Medial vs. combined	0.894	0.756	1.057	0.190
BMI [5-points-OR]	< 0.001		1.097	1.056	1.140	
Defect size	< 0.001	II (4–10 cm) vs. I (< 4 cm)	1.298	1.152	1.462	< 0.001
		III (> 10 cm) vs. I (< 4 cm)	1.343	1.158	1.558	< 0.001
		III (> 10 cm) vs. II (4–10 cm)	1.035	0.915	1.171	0.583
Mesh	0.001	Yes vs. no	0.633	0.480	0.835	
ASA	0.002	III/IV vs. II	1.174	1.059	1.303	0.002
		III/IV vs. I	1.332	1.107	1.603	0.002
		II vs. I	1.134	0.961	1.338	0.137
Gender	0.004	Female vs. male	0.873	0.796	0.956	
Age [10-years-OR]	0.031		0.960	0.925	0.996	
Preoperative pain	0.050	Yes vs. no	1.089	0.984	1.205	0.100
		Unknown vs. no	1.220	1.031	1.444	0.020
		Yes vs. unknown	0.892	0.762	1.045	0.157
Risk factors	0.243	Yes vs. no	1.059	0.962	1.165	
Drainage	0.650	Yes vs. no	1.027	0.914	1.155

**Table 8:** Results of the multivariable analysis for pain at rest in the follow-up including odds ratios with corresponding 95% confidence interval

Variable	*p*-value	Categories	Odds ratio	LCL	UCL	*p*-value (pair-wise)
Age [10-years-OR]	< 0.001		0.822	0.801	0.844	
Preoperative pain	< 0.001	Yes vs. no	1.682	1.555	1.820	< 0.001
		Unknown vs. no	1.413	1.238	1.613	< 0.001
		Yes vs. unknown	1.190	1.055	1.343	0.005
Gender	< 0.001	Female vs. male	1.544	1.444	1.650	
Postoperative complications	< 0.001	Yes vs. no	1.775	1.609	1.958	
EHS classification	< 0.001	Medial vs. lateral	0.674	0.618	0.735	< 0.001
		Lateral vs. combined	1.264	1.099	1.453	< 0.001
		Medial vs. combined	0.852	0.754	0.962	0.010
BMI [5-points-OR]	< 0.001		0.931	0.905	0.958	
Recurrent operation	< 0.001	Yes vs. no	1.180	1.090	1.277	
Drainage	< 0.001	Yes vs. no	1.174	1.075	1.282	
Defect size	< 0.001	III (> 10 cm) vs. I (< 4 cm)	1.227	1.104	1.363	< 0.001
		II (4—10 cm) vs. I (< 4 cm)	1.136	1.043	1.237	0.003
		III (> 10 cm) vs. II (4–10 cm)	1.080	0.989	1.179	0.086
ASA	< 0.001	III/IV vs. I	1.297	1.136	1.481	< 0.001
		II vs. I	1.215	1.082	1.365	< 0.001
		III/IV vs. II	1.067	0.988	1.152	0.099
Mesh	0.151	Yes vs. no	1.198	0.936	1.532	
Risk factors	0.165	Yes vs. no	1.051	0.980	1.127	
Operation technique	0.378	Component separation vs. open—IPOM	1.147	0.966	1.362	0.118
		Open—IPOM vs. open—onlay	0.902	0.783	1.040	0.156
		Component separation vs. open—sublay	1.117	0.954	1.307	0.169
		Open—onlay vs. open—sublay	1.079	0.953	1.222	0.228
		Open—direct suture vs. Component separation	0.841	0.623	1.133	0.255
		Open—direct suture vs. open—onlay	0.870	0.658	1.150	0.328
		Open—IPOM vs. open—sublay	0.974	0.888	1.068	0.573
		Open—direct suture vs. open—sublay	0.939	0.724	1.218	0.634
		Component separation vs. open—onlay	1.035	0.854	1.254	0.727
		Open—direct suture vs. open—IPOM	0.964	0.739	1.257	0.787

**Table 9:** Results of the multivariable analysis for pain on exertion in the follow-up including odds ratios with corresponding 95% confidence interval

Variable	*p*-value	Categories	Odds ratio	LCL	UCL	*p*-value (pair-wise)
Age [10-years-OR]	< 0.001		0.769	0.753	0.785	
Gender	< 0.001	Female vs. male	1.596	1.514	1.683	
Preoperative pain	< 0.001	Yes vs. no	1.616	1.521	1.718	< 0.001
		Unknown vs. no	1.322	1.191	1.468	< 0.001
		Yes vs. unknown	1.223	1.110	1.347	< 0.001
Postoperative complications	< 0.001	Yes vs. no	1.546	1.422	1.680	
EHS classification	< 0.001	Medial vs. lateral	0.698	0.650	0.749	< 0.001
		Medial vs. combined	0.807	0.733	0.889	< 0.001
		Lateral vs. combined	1.156	1.034	1.293	0.011
Defect size	< 0.001	III (> 10 cm) vs. I (< 4 cm)	1.318	1.211	1.433	< 0.001
		II (4–10 cm) vs. I (< 4 cm)	1.204	1.125	1.288	< 0.001
		III (> 10 cm) vs. II (4–10 cm)	1.095	1.020	1.174	0.012
BMI [5-points-OR]	< 0.001		0.945	0.924	0.967	
Drainage	< 0.001	Yes vs. no	1.173	1.094	1.258	
Recurrent operation	< 0.001	Yes vs. no	1.150	1.079	1.226	
Operation technique	0.001	Open—onlay vs. open—sublay	1.187	1.076	1.309	< 0.001
		Open—IPOM vs. open—onlay	0.837	0.748	0.937	0.002
		Open—direct suture vs. open—onlay	0.754	0.606	0.938	0.011
		Component separation vs. open—sublay	1.151	1.014	1.307	0.030
		Open—direct suture vs. Component separation	0.777	0.614	0.984	0.036
		Component separation vs. open—IPOM	1.159	1.009	1.331	0.037
		Open—direct suture vs. open—sublay	0.895	0.730	1.097	0.285
		Open—direct suture vs. open—IPOM	0.901	0.732	1.109	0.324
		Component separation vs. open—onlay	0.970	0.831	1.132	0.698
		Open—IPOM vs. open—sublay	0.994	0.923	1.069	0.862
Risk factors	0.008	Yes vs. no	1.078	1.019	1.140	
ASA	0.020	II vs. I	1.131	1.034	1.236	0.007
		III/IV vs. I	1.148	1.035	1.273	0.009
		III/IV vs. II	1.015	0.954	1.080	0.638
Mesh	0.175	Yes vs. no	1.142	0.943	1.384

**Table 10:** Results of the multivariable analysis for pain requiring treatment in the follow-up including odds ratios with corresponding 95% confidence interval

Variable	*p*-value	Categories	Odds ratio	LCL	UCL	*p*-value (pair-wise)
Age [10-years-OR]	< 0.001		0.787	0.764	0.810	
Preoperative pain	< 0.001	Yes vs. no	1.941	1.770	2.127	< 0.001
		Unknown vs. no	1.670	1.439	1.938	< 0.001
		Yes vs. unknown	1.162	1.017	1.327	0.027
Gender	< 0.001	Female vs. male	1.622	1.504	1.749	
Postoperative complications	< 0.001	Yes vs. no	1.910	1.718	2.125	
EHS classification	< 0.001	Medial vs. lateral	0.676	0.613	0.745	< 0.001
		Medial vs. combined	0.819	0.717	0.937	0.004
		Lateral vs. combined	1.212	1.039	1.415	0.014
Recurrent operation	< 0.001	Yes vs. no	1.286	1.180	1.402	
ASA	< 0.001	III/IV vs. I	1.493	1.283	1.736	< 0.001
		II vs. I	1.307	1.144	1.494	< 0.001
		III/IV vs. II	1.142	1.048	1.244	0.002
Defect size	< 0.001	III (> 10 cm) vs. I (< 4 cm)	1.296	1.152	1.458	< 0.001
		II (4–10 cm) vs. I (< 4 cm)	1.169	1.061	1.287	0.002
		III (> 10 cm) vs. II (4–10 cm)	1.109	1.007	1.223	0.037
Drainage	< 0.001	Yes vs. no	1.211	1.097	1.338	
Risk factors	< 0.001	Yes vs. no	1.153	1.067	1.246	
BMI [5-points-OR]	0.011		0.961	0.931	0.991	
Operation technique	0.023	Open—direct suture vs. open—onlay	0.696	0.517	0.936	0.017
		Open—direct suture vs. component separation	0.694	0.504	0.954	0.025
		Open—onlay vs. open—sublay	1.166	1.017	1.337	0.028
		Open—direct suture vs. open—IPOM	0.757	0.571	1.003	0.052
		Component separation vs. open—sublay	1.169	0.983	1.390	0.077
		Open—direct suture vs. open—sublay	0.811	0.616	1.069	0.137
		Open—IPOM vs. open—sublay	1.072	0.968	1.187	0.184
		Open—IPOM vs. open—onlay	0.919	0.786	1.074	0.289
		Component separation vs. open—IPOM	1.091	0.904	1.317	0.365
		Component separation vs. open—onlay	1.003	0.812	1.238	0.980
Mesh	0.388	Yes vs. no	0.893	0.691	1.154	

### Standardized differences for patients with and without follow-up

The results of the standardized differences for patients with (*n* = 39,523) and without (*n* = 22,361) follow-up verified that there was no bias in the patient selection of the analysis population. Patients in the analysis population were on average 3.3 years older, received more often a mesh and were less frequently operated with direct sutures. The standardized difference was above the reference value of 10%. For all other variables, including the complication rates, standardized differences of less than 0.1 were found, thus indicating no bias in patient selection.

## Discussion

Should surgeons in case of doubt use drains or not in elective open incisional hernia surgery? It is beyond dispute that surgical drains help to remove access fluid which is assumed to reduce wound-related complications and seroma formation, but these advantages may nevertheless be counterbalanced with certain downsides like an increased risk of infections and postoperative pain. To shed more light on this question, we performed a Herniamed registry-based evaluation of prospectively collected data of 39,523 patients which is so far the most comprehensive quality assurance study in Germany. The influence of drains on the outcome of hernia operations has already been examined in several controlled randomized trials and meta-analyses in the past [10, 11]. A registry analysis, however, enables an analysis of the clinical results as part of health services research and points out possible differences between the current status of research and patient care in clinical routine. This analysis of a large clinical data basis is thus an important contribution to understand the “real world” effect of a treatment outside the tightly controlled environment of randomized trials [8].

Our investigations covered the period from 2009 to 2020 with 39,523 elective open incisional hernia operations, during which 28,182 patients (71.31%) received a drain. The high frequency of drain use in more than 2/3 of the patient collective clearly mirrors the high acceptance of drainages in clinical routine. The unadjusted analysis of the relationship between drain use, patient variables, and operation characteristic shows that the expression of almost all features differed significantly. Only with respect to the gender, no difference was observed. Drained patients had a significantly higher age (63.6 vs. 59.8, *p* < 0.001), higher BMI (29.8 vs. 27.9, *p* < 0.001), higher ASA score (*p* < 0.001), larger hernia defects (*p* < 0.001), and required significantly more frequently mesh application (92.9% vs. 64.6%, *p* < 0.001). All in all, the clinical care situation in the drainage group shows a negative selection with regard to patient and hernia characteristics. The use of drains is typically linked with the more complex operations. In component separation, 89.8% of patients received drain, in open sublay 83.5% and in open onlay 81.8%.

Most studies analysing the influence of drains investigated similar outcome criteria like local complications, particularly bleeding and seroma formation, surgical site infections (SSI), surgical site occurrences (SSO) and surgical site occurrences requiring procedural interventions (SSOPI) [3, 10, 12, 13]. All of these studies point to the fact that single influencing factors are difficult to extract since complications in elective open incisional hernia surgery are caused by numerous parameters, which was also the case in our study. We carried out eight multivariable analyses (intraoperative complications, general complication, postoperative complications, complication-related reoperations, recurrence on 1-year follow-up, pain on exertion at 1-year follow-up, pain on rest at 1-year follow-up, pain requiring treatment at 1-year follow-up). With the exception of recurrences in the follow-up, the use of drains was in each case associated with a significantly higher incidence of complications and higher pain rates. The multivariable analyses also showed a significant association of defect size, ASA and EHS classification in all cases, and for the items operation technique and age in seven of the eight analyses performed.

Placing the focus on subject-specific criteria for drain use such as the influence of local complications, the data situation remains quite heterogeneous in the literature and reveals no clear evidence of a protective effect of drains on seroma formation. Miller et al. compared the outcome of 580 patients each with or without drainage, similar hernia size and robotic surgery with respect to seroma formation at 30 days and found a significantly decreased postoperative seroma occurrence of 3.8% in the group with drainage vs. 15.2% in the group without (*p* < 0.0001) [12]. No significant difference with respect to the use of drains observed Westphalen et al. [11] who assessed the seroma frequency in 21 patients per group with non-significant hernia defect size difference and the exclusion of ASA III–IV patients at three different postoperative ultrasound (US) time points and with seroma frequencies between 19.0 and 52.4% with drain vs. 28.6–57.1% without drain (*p* = 0.469 for early postoperative US; *p* = 0.852 for late US). In a RCT by Willemin et al. [10], fluid collection at 30 days was reported in 60.3% of the drain group patients vs. 62.0% (*p* = 0.844) without drain after open mesh repair, indicating that drains failed to reduce the rate of postoperative fluid collections that might contribute to seroma formation. In our analysis of the clinical care situation with negative selection of the patient population and hernia characteristics as well as more complex hernia surgeries, we observed significantly more seromas when drains were used, even though the rate of seroma formation was generally low (4.8 vs. 2.2% without drain, *p* < 0.001).

In addition to SSOs like seroma formation, also the effect of drain use on SSIs and SSOPI was investigated as decisive factor. Several studies suggest that the use of drains increases the risk of SSIs, while others found no significant difference in infection rates with or without drains. This became particularly evident in data of the Americas Hernia Society Quality Collaborative [12, 13] and in a recent RCS reporting comparable site infection rates in both groups [10]. Westphalen et al. reported no significant difference with or without drain use concerning surgical wound infections [11]. Even in the most recent literature, the data situation shows a heterogeneous picture. In a meta-analysis of ventral hernia repair by Mohamedahmed et al. (2023), drained patient groups had higher SSI rates and longer total operation times in eight studies involving 2568 patients, but no significant advantage was seen in terms of wound-related complications [14]. Marcolin et al. (2023) published a meta-analysis for retromuscular ventral hernia repair with four studies involving 1,724 patients and found no differences in SSI, hematoma, SSO, or SSO-requiring procedural intervention, but the group with drain placement had significantly fewer seromas [15]. Our evaluation of the care situation, however, revealed a significant difference in the patient group with drain vs. without concerning SSI (1.7% vs. 0.7%, *p* < 0.001) and SSO (3.2% vs. 1.3%, *p* < 0.001). In addition, the complication-related reoperation rate was significantly increased when drains were used (*OR* = 1632 [1385; 1924]).

Relationships not evaluated in our analysis are the influence of the time point of drain removal or the prolonged prophylactic use of antibiotics on the SSI and SSO. Plymate et al. showed a linear, non-significant increase of SSO depending on the drain duration [16]. Only a BMI of > 35 represented a predictor of wound occurrence in their study. Other authors found only little persuasive evidence for a prolonged antibiotic use to reduce SSI and SSO [17, 18].

Drains were used in 71.3% of elective open incisional hernia operations between 1/2009 and 12/2020 which indicates a high level of acceptance in the clinical care situation. We assume that a less favourable risk profile of patient and hernias characteristics leads to a negative selection when drains are used. In the following, a significant association with a higher risk of complications and pain is observed for all target parameters with the exception of recurrences. Similar results were also reported in a registry-based multivariable analysis by Schaaf et al. who observed more intraoperative complications, general complications, and complication-related reoperations in patients with drains. In their study, also larger defect size and BMI were unfavourably associated with postoperative complications, recurrences and pain [19]. From a clinical point of view, it is difficult to extract the separate effect of drainages on the complications, as the multivariable analyses showed that these were significantly influenced in all outcome measures by numerous other variables such as defect size, ASA classification, and EHS classification. Apparent in our analysis became however that the use of drains is significantly associated with a higher occurrence of SSI and SSO in the clinical routine, especially if patients with higher BMI and larger defects are concerned.

Taken together, drains are currently used in over 70% of elective open incisional hernia surgeries, based on various criteria such as the complexity of the procedures, hernia characteristics, or patient constitution. Despite adjusting for other influencing variables in the model (independent of patient and surgical characteristics), we observed a significant association between outcomes and drain usage. The poorer patient outcomes are associated with the use of drains, regardless of other factors in the model such as patient or surgical characteristics. However, the use of drains may serve as a surrogate parameter for other unobserved confounding factors. These results should prompt a re-evaluation of the predominantly "traditional" use of drainage and encourage careful case-by-case assessment. Further investigations are required as the data situation still remains heterogeneous.

## Limitations

**Fig. 2:**
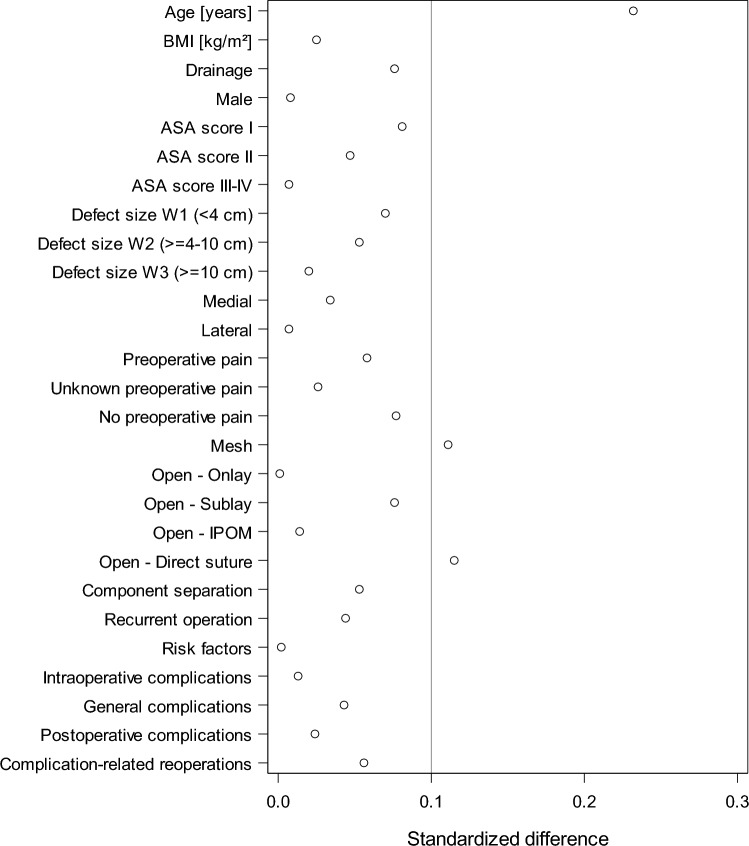
Standardized differences for patients with and without follow-up

Our study has a number of important strengths. The data used in this article are the largest quality-assured data pool in Germany, Austria and Switzerland covering the period from 2009 to 2020; the statistical power to detect changes is thus high. In general, it should be noted that effects that have been proven to be significant do not necessarily have to be also clinically relevant, since even very small differences can be statistically significant due to relatively large number of cases. A limitation of this study is the rate of missing follow-up examinations. In accordance with the selection criteria of the Herniamed registry (see flowchart in Fig. 1), patients with non-incisional hernias, entry-state-key incomplete, operations performed using laparoscopic technique, patients under 16 years of age, emergency operations, patients with physiomesh or operation dates after December 31, 2020, and patients without 1-year follow-up were excluded. The lack of follow-ups (drop out) for a relevant proportion is another limitation of the registry, but the subgroup analysis does not show any selection bias (Fig. 2).

Our analysis was a project in clinical health services research.
